# Structure and Performance Analysis of Signal Acquisition and Doppler Tracking in LEO Augmented GNSS Receiver

**DOI:** 10.3390/s21020525

**Published:** 2021-01-13

**Authors:** Li Cheng, Yonghong Dai, Wenfei Guo, Jiansheng Zheng

**Affiliations:** 1 School of Electronic Information, Wuhan University, Wuhan 430079, China; lcheng996@gmail.com (L.C.); yhdai@whu.edu.cn (Y.D.); zjs@whu.edu.cn (J.Z.); 2 GNSS Research Center, Wuhan University, Wuhan 430079, China

**Keywords:** acquisition, Doppler shift, GNSS receiver, LEO satellite, weak signal tracking

## Abstract

Due to the low signal power, the Global Navigation Satellite System (GNSS) signal is vulnerable to interference and even cannot be captured or tracked in harsh environments. As an alternative, the Low Earth Orbit (LEO) satellite has been widely used in the navigation field due to the advantages of low cost and strong signals. It is becoming a significant component of the new combined navigation system with GNSS. The combination of an LEO Doppler signal and GNSS observables can improve the positioning accuracy and high-precision positioning convergence time of the GNSS receiver. However, the GNSS signal receiving capability cannot be improved from this data fusion level. We propose a novel assisted structure where GNSS signal acquisition and Doppler tracking are assisted by LEO Doppler positioning. The receiver uses the LEO signal to achieve Doppler positioning firstly. Then, the coarse position with the GNSS navigation messages received from LEO, as well as the estimated clock information, is used to assist in the acquisition and tracking of GNSS. In this way, the GNSS receiver’s sensitivity can get the benefit from this integrated system. The paper presents the structure of the assisted receiver and analyzes the assisted GNSS signal acquisition and carrier tracking performance in detail. Simulation experiments of this assisted structure are carried out to verify its superiority of acquisition and tracking sensitivity in comparison with standalone GNSS receivers. Theoretical analysis and experimental results show that the proposed acquisition method can achieve 90% detection probability at a carrier-to-noise ratio (C/N_0_) of 15 dB-Hz, which is about 8 dB higher than the conventional acquisition method without assistance; the proposed tracking method can track weak signals of *5* dB-Hz, which is about 4 dB higher than the conventional method. Therefore, this novel LEO-assisted receiver has significantly improved weak signal acquisition and tracking sensitivity.

## 1. Introduction

Due to the high satellite orbit, the Global Navigation Satellite System (GNSS) has low signal landing power and is susceptible to interference. Many works investigate various signal-processing methods to acquire and track weak signals. For weak GNSS signal acquisition, a block averaging pre-processing (BAP) approach that coherently accumulates signal blocks to improve the signal power is introduced [1]. To solve the bit transition problem in coherent integration, block accumulating semi-coherent integration of correlations (BASIC) was proposed in 2008 [2]. In addition to acquisition, weak signal tracking is also important, for the tracking loop is necessary to extract the observables. To track a signal under dynamic stress and Radio Frequency interference (RFI), Ward proposed an FLL-assisted-PLL tracking method, which integrates both the frequency-locked loop (FLL) and the phase-locked loop (PLL) characteristics and obtains the best overall performance [3]. Lian [4] puts forward a Kalman filter-based tracking algorithm and applies a wavelet de-noising technique in the PLL, which demonstrates that the strategies work well under low signal-to-noise ratio (SNR) and high dynamic applications. For a high sensitivity receiver, no matter whether in acquisition or tracking, the key problem is to extend the coherent time [5].

To further improve the availability and sensitivity of the Global Navigation Satellite System, augmentation from the other system is always used, including internet ephemeris and receiver Doppler information. If the receiver can get the ephemeris information in another way, FLL can be used to keep the loop on track and extract the observables, which has a higher sensitivity than PLL. Satyanarayana [6] proposes a new architecture for Global Positioning System (GPS) weak signal frequency tracking, which uses squaring and block processing to track signals with a carrier-to-noise ratio (C/N_0_) as low as 14.5 dB-Hz under static conditions. Chiou designed a Doppler-aided GPS navigation system for weak signals in his dissertation, which mainly uses the FLL to track the signal under strong ionospheric scintillation [7]. Tao gives a comprehensive lecture on how an FLL works in the weak signal environments of high sensitivity tracking and compares the performance of different FLL tracking methods [8]. Our previous work [9] designs a double-stage Numerically Controlled Oscillator (NCO)-based tracking loop with a Differential Amplitude (DA) frequency discriminator, which has absolute linearity in a wide pull-in range and can improve the carrier tracking sensitivity of the GNSS receiver in a weak signal environment. However, the A-GNSS system has its limitations in the forest area, since the internet is not available. Moreover, it cannot provide the receiver status to help receive the GNSS signals in harsh environments. As for the high dynamic condition, the most widely used technology is GNSS/Inertial Navigation System (INS) deep integration, which could eliminate the Doppler in the tracking loop with receiver speed estimated from the Inertial Measurement Unit (IMU) and satellite speed obtained from ephemeris [10,11]. This integration method is very suitable for high dynamic or strong interference conditions [12]. The main shortcomings are that the IMU could not provide a good Doppler estimation for the receiver when it is static [13]. Neither Assisted-GNSS (A-GNSS) nor a GNSS/INS coupled system can provide the clock information, which may become the main error factor in a long coherent integration [14,15].

Low Earth Orbit (LEO) satellite systems, most of which are designed for communicating, have higher received power and faster-moving velocity than GNSS, for they are running at the lower orbit. Due to these characteristics, LEO has been recently used for positioning or augmenting the GNSS system [16,17]. However, these augmentations are still on the system or observable level, which could not improve the GNSS signal received ability, especially under harsh conditions. As for a standalone positioning system, the LEO signal could be used to realize positioning by its Doppler frequency as a Signal of Opportunity (SoOP) [18,19]. Moreover, LEO satellites have the potential to transmit navigation signals [20]. Nowadays, it is also considered to transmit the Pseudorandom Noise (PRN) signal as GNSS system to get a more precise position [21]. Given these functions, the GNSS receiver can use the position information from LEO positioning to estimate the Doppler of the GNSS signal. Moreover, by the navigation signals transmitted by the LEO satellite, the receiver can extend the coherent integration time and further compress the loop bandwidth.

A novel LEO augmented GNSS system and receiver structure is proposed in this paper so that the augmentation to the signal processing can be explored. In this designed system, LEO transmits GNSS ephemeris through its communication channel. Meanwhile, it can be used to locate the ground receiver by its Doppler frequency. In weak signal environments, the receiver realizes positioning using the LEO signal by Doppler shift first. The receiver could also obtain GNSS ephemeris information from the LEO system. Then, the ephemeris, as well as the estimated location and Doppler from the LEO satellite is used to reduce the GNSS signal searching range and extend the coherent integration time to capture weak signals in the acquisition stage. Meanwhile, the receiver clock bias and drift could be estimated in the LEO positioning. With this information, the receiver could achieve longer coherent integration and narrower loop bandwidth to reduce the noise and track the weak signal. Unlike the GNSS/INS deep coupling technology, LEO communication satellites can locate the receiver autonomously, estimate the basic position of the GNSS receiver, and provide much more aiding information, including ephemeris, navigation data, Doppler shift, and clock information assisting for the acquisition and tracking of the GNSS signal. In this contribution, we describe the new assisted acquisition and tracking scheme, with the analysis and verification of its performance by the simulation results.

The paper is organized as follows: Section 2 introduces the LEO-assisted GNSS receiver structure and describes the signal acquisition and tracking method. Section 3 analyzes three types of errors from the LEO satellite assistance. Section 4 gives the simulation results of the assisted acquisition and tracking performance. A verified platform is realized in Section 5, and the GPS signal is simulated to test the performance of this structure, which is compared with an unassisted system to show the advantages. Finally, conclusions are drawn in Section 6.

## 2. Novel LEO Augmented GNSS Receiver System Structure

**Figure 1:**
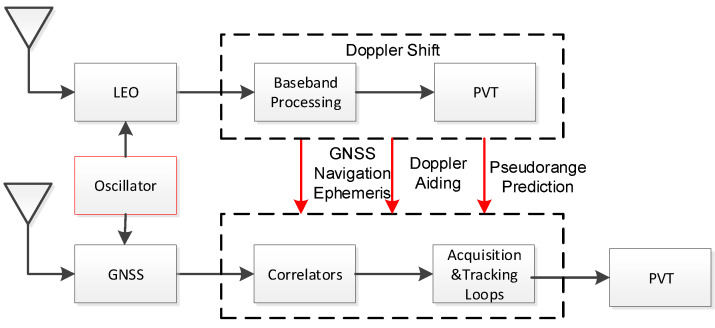
Low Earth Orbit (LEO)-augmented Global Navigation Satellite System (GNSS) receiver system structure.

To solve the problem of the low sensitivity of GNSS receiver acquisition and tracking under weak signal conditions, we propose a novel LEO augmented system and receiver structure. The proposed system differs from the other LEO augmented system in transmitting the GNSS ephemeris to receivers. As shown in Figure 1, the receiver includes both LEO and GNSS antennas and RF front ends, and the two RF parts share the same oscillator. The receiver extracts Doppler information and GNSS satellite ephemeris information transmitted by LEO satellites through baseband processing of low-orbit satellite signals. The initial positioning of the receiver can be estimated by the Doppler information. Then, the receiver assists the baseband processing of the GNSS signal through the initial position and the received information. In the implementation, the receiver uses the position information and the GNSS satellite ephemeris to calculate the line of sight (LOS) between the receiver and the GNSS satellite. With the help of estimated pseudo-range and Doppler information, the frequency and code search intervals of the GNSS signal can be greatly reduced, which means that the acquisition efficiency can be improved. On the other hand, the receiver can estimate the initial clock frequency using the Doppler positioning algorithm. The receiver takes the orbit, time, Doppler shift, oscillator, and GNSS navigation message as auxiliary information to assist in extending the coherent integration time, thereby effectively compressing the bandwidth and improving the sensitivity of acquisition and tracking. This will eventually improve the positioning performance in the GNSS weak signal environments.

### 2.1. Acquisition Method in the LEO-Augmented GNSS Receiver

The digitized Intermediate Frequency (IF) signal received at the end of the GNSS receiver RF front-end can be presented as:

(1) $$r\left(i\right)=A\left(iT_{s}\right)D\left(iT_{s}\right)C\left(iT_{s}-\tau \right)\cdot cos\left[2\pi \left(f_{IF}+f_{d}\right)T_{s}i+\phi \right]+n\left(iT_{s}\right)$$

where r(i) is the received IF GNSS signal at the end of the RF front-end sampled at time $t=iT_{s}$. $T_{s}$ is the sample period. $A\left(iT_{s}\right)$ is the signal amplitude. $D\left(iT_{s}\right)$ represents the navigation data bits. $C\left(iT_{s}-\tau \right)$ is the spreading code sequence for GNSS, and τ is the code delay in samples. $f_{IF}$ is the nominal Intermediate Frequency (IF) in Hertz. $f_{d}$ is the carrier Doppler frequency shift in Hertz. ∅ is the carrier phase in rad/s. $n\left(iT_{s}\right)$ is the additional band-limited white Gaussian noise. The bilateral power spectral density of the white Gaussian noise is $N_{0}/2$. The purpose of the signal acquisition is to estimate the code shift τ and carrier Doppler shift $f_{d}$. The receiver needs to perform a two-dimensional search of the maximum range of Doppler frequency and initial code phase for each satellite. In addition, because of the navigation bit reversal, the coherent integration time of a conventional receiver is limited to 10 ms.

**Figure 2:**
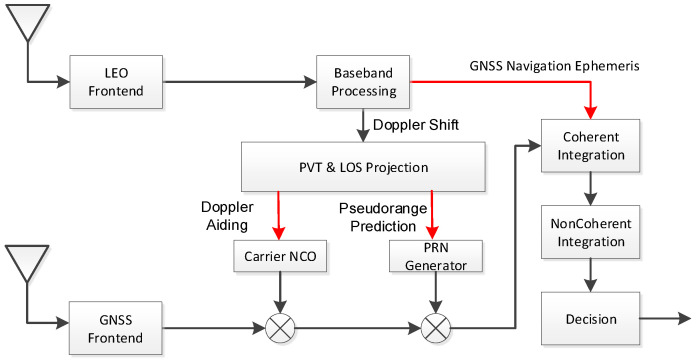
LEO-assisted GNSS signal acquisition.

We propose a method of LEO positioning-assisted GNSS signal acquisition to improve the acquisition efficiency and sensitivity. As shown in Figure 2, the baseband processing block estimates the Doppler shift from the LEO satellite. The initial position of the GNSS receiver can be predicted through the LEO Position, Velocity, and Time (PVT) solution algorithm. After projection on LOS, the Doppler and pseudo-range in GNSS can be estimated and used as the reference information of the carrier NCO and PRN generator respectively. Thus, the acquisition efficiency of the receiver can be improved by the Doppler and pseudo-range information. Moreover, the bit inversion problem in the GNSS signal can be eliminated by the navigation message received from the LEO satellite. Therefore, the acquisition sensitivity of the receiver can be effectively improved by the extended coherent integration time.

### 2.2. Tracking Method in the LEO-Augmented GNSS Receiver

Signal acquisition can be considered as a coarse estimation of GNSS signal parameters, which is unable to meet positioning requirements. On the other hand, the relative motion between the satellite and the receiver causes the signal parameters to change over time. Carrier and code tracking loops with feedback functions are often used as estimates of fine GNSS signal parameters in the tracking stage. The rate of the PRN code is much lower than the carrier frequency. Thus, the carrier-tracking loop is so fragile to be the most important part of a GNSS receiver. Under weak signal conditions, the loop bandwidth is often reduced to improve the accuracy of the carrier tracking loop. However, as a result of the oscillator noise and dynamics on the carrier tracking loop, the bandwidth cannot be reduced arbitrarily. Otherwise, it will cause the phenomena of lock-lose.

**Figure 3:**
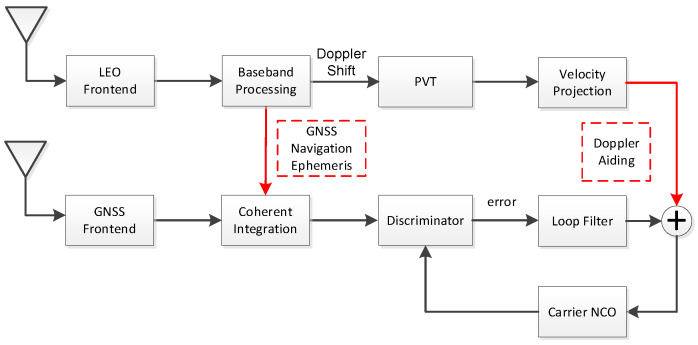
LEO-assisted carrier tracking loop structure.

To improve the tracking ability of the receiver in weak signal environments, such as strong wideband jamming in the military, this paper proposes a carrier tracking loop structure that is LEO satellite-assisted. It is noteworthy that the receiver does not need to extract the navigation bit from the signal processing, for it can get the GNSS ephemeris from LEO. On the other hand, the receiver is supposed to work well when it starts to encounter the jamming stress. At this time, the local PNR code is still aligned to the received signal and can be used to assist the loop with ephemeris. The navigation bit is used to remove the bit reversal and extend the coherent integration time. Therefore, only FLL is considered to track the ultra-low SNR signals. As shown in Figure 3, the receiver uses the obtained satellite navigation ephemeris to remove the bit reverse in the coherent integration of the GNSS signal after receiving and demodulating the signal sent by the LEO satellite. It can effectively extend the time of coherent integration and improve the input SNR of the loop. Furthermore, the receiver obtains the local initial position by Doppler positioning from the LEO signal. The receiver uses the position information and GNSS ephemeris information to calculate the Doppler of the GNSS signal, which can eliminate the dynamic effects and further compress the loop bandwidth. As a result, the tracking sensitivity can be enhanced.

**Figure 4:**
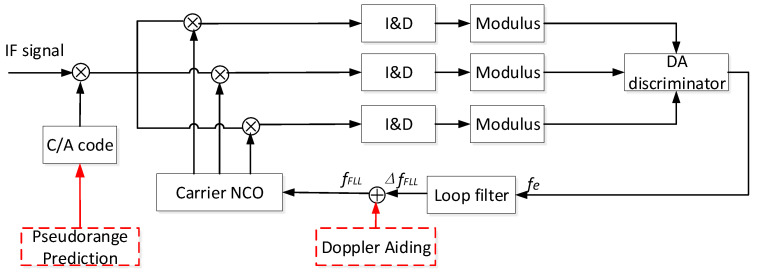
Frequency-locked loop (FLL) structure based on a DA frequency discriminator.

To track GNSS signals with ultra-low SNR, we use a frequency-locked loop (FLL) to improve the loop’s tracking ability. The commonly used Costas discriminator has a pull-in range between $-1/\left(2T_{coh}\right)$ and $1/\left(2T_{coh}\right)$. When the integration time is long enough, the oscillator frequency drift and the Doppler rate-of-change could cause the frequency error to exceed the pull-in range. In high dynamics, a Differential Amplitude-based frequency discriminator [9] with a wider frequency pull-in range is adopted in this paper. As shown in Figure 4, the local carrier is divided into three channels (fast frequency, slow frequency, and estimated frequency) which correlate with the signal removed by pseudocode. The results of the three-channel correlators are accumulated after coherent integration and non-coherent integration. Then, these results enter the Differential Amplitude discriminator for frequency discrimination. The frequency error $f_{e}$ processed by the loop filter is used with the Doppler shift from LEO satellite to adjust the carrier NCO.

In Figure 4, we assume that the signal in Equation (1) removed the PRN code correctly. Thus, an obtained complex signal satisfies the following expression:

(2) $$r\left(t\right)=AD\left(t\right)\left[cos\left(2\pi f_{e}t+\phi_{e}\right)+jsin\left(2\pi f_{e}t+\phi_{e}\right)\right]+n\left(t\right).$$

Ignoring high-frequency information items, the output of the integration and dump circuit by integrating the signal within a bit interval can be written as [22]:

(3) $$s\left(t\right)=AD\left(t\right)sinc\left(\pi f_{e}T_{coh}\right)e^{j\left[2\pi f_{e}\left(t+\frac{T_{coh}}{2}\right)+\phi_{e}\right]}+n\left(t\right)$$

where $T_{coh}$ represents the coherent integration time, and the sinc(x) function equals sin(x)/x. In a single bit, the Differential Amplitude (DA) frequency discriminator estimates the standard frequency error $f_{e}$ by the following equation:

(4) $$f_{e}=\Delta f\cdot \frac{A_{s}-A_{f}\;}{\left[A_{s}-2cos\left(\pi \Delta fT_{coh}\right)A_{p}+A_{f}\right]}.$$

In Equation (4), $A_{s}=\left|Asinc\left(\left(f_{e}-\Delta f\right)T_{coh}\right)\right|$, $A_{f}=\left|Asinc\left(\left(f_{e}+\Delta f\right)T_{coh}\right)\right|$, and $A_{p}=\;\left|Asinc\left(f_{e}T_{coh}\right)\right|$. Δf stands for the interval of three local carrier frequencies. When $\Delta f=2/\left(3T_{coh}\right)$, this frequency discriminator can obtain the optimal frequency estimation error and reach the lowest tracking threshold. After the frequency error $f_{e}$ is calculated, it is filtered through a second-order loop filter, which finally is fed back through the carrier NCO. Significantly, in the LEO-assisted tracking loop, the navigation bit *D*(*t*) in Equation (3) could be removed according to the information from the LEO, resulting in a longer integration time than a bit duration.

## 3. Error Introduced by LEO Satellite Assistance

To determine the parameters of acquisition and tracking and analyze the performance of LEO positioning assistance, it is necessary to consider the errors introduced by the assistance information from the LEO satellite. These errors include the clock accuracy of the satellite and receiver, the clock stability error of the satellite and receiver, and the Doppler deviation caused by the position error introduced by the positioning error from LEO satellite. The Doppler deviation and the clock accuracy of the receiver mainly affect the acquisition of a GNSS signal. A larger deviation increases the search range, resulting in low efficiency. On the other hand, the clock stability of the receiver prevents excessive compression of the coherent integration time. This section will analyze each error in detail. In the analysis, the receiver is assumed to be static. In the analysis and simulation, GPS constellation is considered for GNSS. Moreover, the parameters for LEO positioning are mainly derived from the Iridium satellite, whose positioning technology is mature enough. The only difference is that the LEO is assumed to transit GNSS ephemeris in this work. GPS satellites follow near-circular orbits at about 20,200 km altitude, and Iridium uses 66 spacecraft orbiting at an altitude of 780 km, which is much lower than the GPS orbit altitude [23,24]. The velocities of a GPS satellite and an Iridium satellite relative to the earth are about 3862 m/s and 7500 m/s, respectively. The GPS L1 C/A signal uses a frequency of 1575.43 MHz, while Iridium uses the frequency 1624 MHz.

### 3.1. LEO Positioning and Timing Error

**Figure 5:**
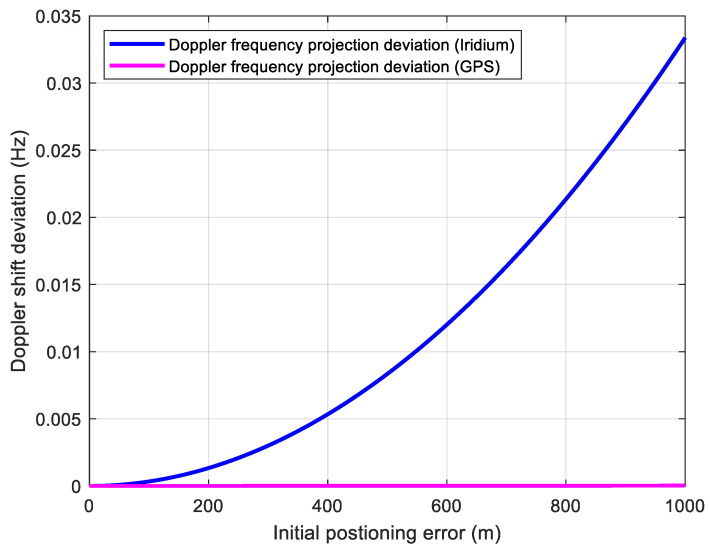
Doppler shift deviation caused by initial positioning error.

For a static receiver, taking the Iridium satellite as an example, the position error obtained by LEO satellite through Doppler positioning is within 1 km, and the timing accuracy is about 1 μs [19]. It is obvious that the influence of timing error is within one code chip for GPS. However, for the positioning error, an error of 1 km corresponds to three code chips. The position error of the receiver affects not only the search range of the receiver’s code phase but also the accuracy of the estimated Doppler shift caused by the satellite dynamic. The relative movement between the receiver and the satellite causes the Doppler shift of the satellite signal. Under the assumption that the receiver is stationary, the Doppler shift is mainly caused by satellite motion. In the process of positioning solutions using LEO and GNSS signals, the receiver could estimate Doppler shifts through signal processing correctly. However, the initial position deviation of the receiver will cause errors in the estimated Doppler projection to the GNSS signal LOS. This frequency error caused by the projection satisfies the following expression:

(5) $$f_{e}=v\times \left(1-cos\left(\frac{Z}{H}\right)\right)\times \frac{f_{sat}}{c}$$

where *v* and H represent the orbital speed and altitude of the satellite respectively. Z indicates the position error of the receiver. c is the speed of light. For LEO-assisted GNSS receivers, the estimated Doppler deviation of LEO signal after projection will be absorbed into the receiver clock frequency deviation. However, the Doppler frequency shift of the GNSS signal will be affected by the position error after LOS projection. Figure 5 shows the effect of receiver position error on Doppler shift estimation. It can be seen from Figure 5 that the Doppler estimation errors caused by the position error on Iridium and GPS dynamics are both small enough to be ignored.

### 3.2. Clock Accuracy of Sattelite and Receiver

In addition to the relative movement between the satellite and the receiver, the oscillator frequency bias of the satellite and receiver also affects the measurement of the received carrier Doppler. It can be obtained as follows:

(6) $$f_{CLK\_e}=\gamma \times f_{carr}$$

where γ represents the frequency accuracy of the clock. $f_{carr}$ indicates the carrier frequency of the GNSS signal. The accuracy of the clock frequency will affect the receiver’s Doppler search. The deviation of the clock frequency includes two parts: the satellite clock and the receiver clock. The LEO satellite clock’s accuracy is about $1\;\times \;10^{-9}$, which could cause the Doppler error at about 1.58 Hz. When the receiver uses the LEO satellite for positioning, it absorbs the clock frequency deviation of the LEO satellite into the receiver’s clock. The deviation of the receiver’s clock frequency accuracy will also cause the Doppler error to deviate from the true value. The GPS receiver clock frequency accuracy is about $5\;\times \;10^{-9}$. Then, the introduced Doppler frequency shift error can be calculated to be about 7.88 Hz. Therefore, the Doppler shift error caused by the clock accuracy of LEO satellite and the receiver is within 10 Hz.

### 3.3. Clock Stability of Sattelite and Receiver

In addition to the clock accuracy, the clock stability of the satellite and receiver can also cause the frequency bias, resulting in signal attenuation when a long coherent integration is adopted. The frequency stability of the clock refers to the relative change in frequency caused by the oscillator noise. This noise is usually expressed in the form of power-law noise, and its power spectral density can be expressed as:

(7) $$S_{y}\left(f\right)=h_{\alpha }f^{\alpha }$$

where f represents frequency, $h_{\alpha }$ indicates the intensity factor, and α is the index of power-law noise. When α=0, α=−1, and α=−2, it stands for white noise, flicker noise, and random walk noise, respectively. In applications, the Allan variance is often used to evaluate the time-domain frequency stability of the frequency. It can be determined by [25]:

(8) $$\delta_{y}^{2}\left(\tau \right)=\frac{S_{f}}{\tau }+\frac{S_{g}\tau }{3}.$$

$S_{f}$ and $S_{g}$ indicate the phase and frequency drift strength of the clock. $S_{f}\sim h_{0}/2$, $S_{g}\sim 2\pi^{2}h_{-2}$.

**Figure 6:**
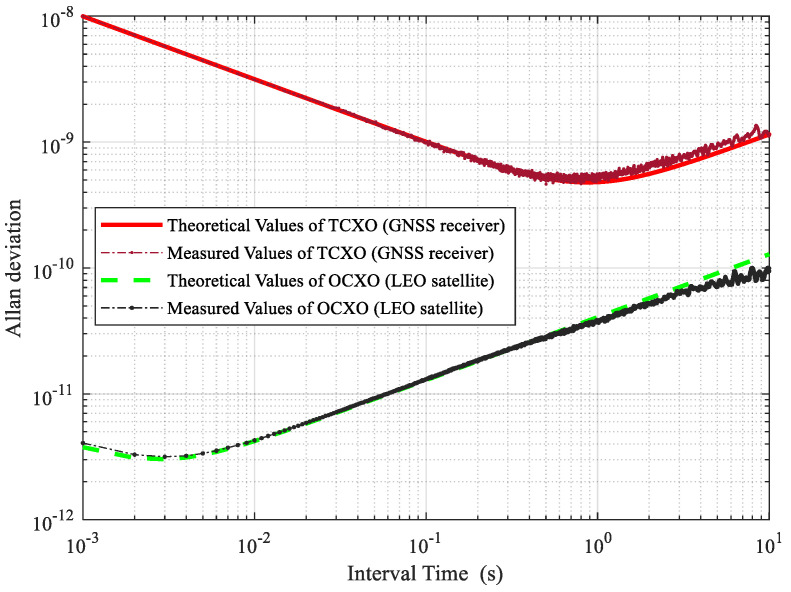
Theoretical and measured clock Allan variances.

The frequency stability of the clock is related to the selected oscillator type. In general, LEO satellites often choose the Oven Controlled Crystal Oscillator (OCXO) with better stability [23], while GNSS receivers often use the Temperature Compensated Crystal Oscillator (TCXO) with general performance. In the LEO-assisted GNSS system, when the SNR is improved through a long coherent integration time, the frequency deviation caused by the clock instability can result in the attenuation of the integrated signal. To analyze the influence of clock noise, this paper uses OCXO and TCXO typical parameters to generate clock noises for simulation of LEO satellites and receivers, respectively. In the simulation, the parameters of the TCXO are set to be $h_{0}=2\times 10^{-19}$, $\;h_{-2}=2\times 10^{-20}$, and the parameters for the OCXO are chosen as $h_{0}=2.51\times 10^{-26}$, $h_{-2}=2.51\times 10^{-22}$. The clock model used in the simulation is as follows:

(9) $$\left[\begin{array}{c} \delta t\left(k+1\right)\\ \delta f\left(k+1\right) \end{array}\right]=\left[\begin{array}{cc} 1 & \Delta t_{k}\\ 0 & 1 \end{array}\right]\left[\begin{array}{c} \delta t\left(k\right)\\ \delta f\left(k\right) \end{array}\right]+\left[\begin{array}{cc} \sqrt{S_{f}\Delta t_{k}+S_{g}\Delta t_{k}{}^{3}/3} & \sqrt{S_{g}\Delta t_{k}{}^{2}/2}\\ \sqrt{S_{g}\Delta t_{k}{}^{2}/2} & \sqrt{S_{g}\Delta t_{k}} \end{array}\right]w_{k}$$

where $\Delta t_{k}$ is the time interval, and $w_{k}$ is an identity matrix. Figure 6 is the Allan deviation of the simulated clock noise sequences of OCXO and TCXO. It can be seen from the figure that the performance of the clock generated by this clock model is consistent with the theoretical analysis. The Allan deviation of OCXO caused by the oscillator noise is not higher than $10^{-10}$ within 10 s. The stability of TCXO reaches its lowest value around 500 ms. As the time interval is further lengthened, the frequency deviation caused by noise begins to increase gradually.

**Figure 7:**
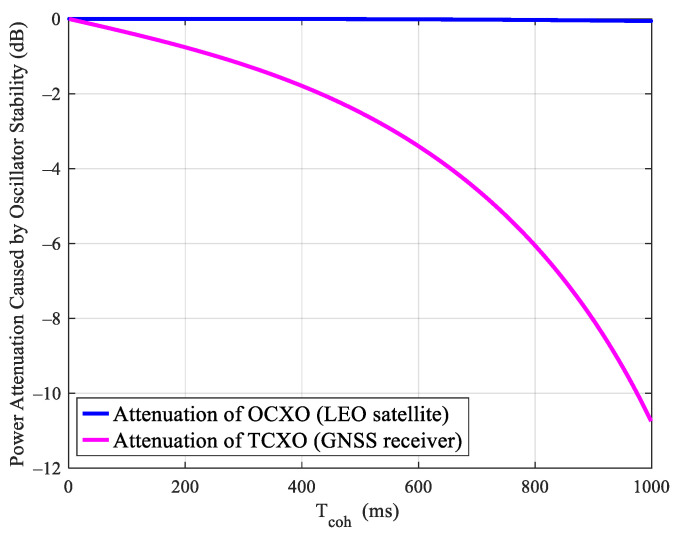
Power attenuation caused by oscillator stability.

When Doppler positioning is performed on a GNSS receiver through an LEO satellite, the GNSS receiver will absorb the LEO satellite’s clock error. Therefore, the impact of the LEO satellite’s clock and GNSS receiver’s clock on signal processing should be considered at the same time. For the GNSS receiver, the clock frequency error caused by instability will cause the attenuation of the coherent gain. The power attenuation caused by coherent integration can be given by:

(10) $$G_{d}=10lgsinc^{2}\left(f_{e}T_{coh}\right)$$

where $f_{e}$ is the frequency deviation, and $T_{coh}$ represents the coherent integration time. The frequency attenuation of different crystal oscillator types under different coherent integration times can be obtained in combination with Figure 6, which is shown in Figure 7. It can be seen from this figure that as the LEO satellite uses OCXO, the impact of clock noise can be negligible under the coherent integration time within 1 s. Since the GNSS receiver adopts the TCXO oscillator, the signal power attenuation increases along with the coherent integration time. That is the reason why the integration time cannot be compressed indefinitely. It can be seen from the figure that to make sure the gain attenuation is no more than 2 dB, the maximum coherent integration should not exceed 400 ms. Furthermore, it is obvious that a receiver using OCXO can use a longer coherent integration, which could achieve several seconds when an ultra-stable clock is used [14,15]. However, the motion and non-linearity of the receiver should be considered carefully for such a long coherent integration. Thus, we just take the most common TCXO as the clock of the receiver in this work.

## 4. Augmented Performance Analysis

The purpose of the LEO-assisted GNSS structure is to improve the sensitivity of GNSS signal acquisition and tracking. With the assistance of LEO, the influence of navigation bit reversal is eliminated. Then, the coherent integration time can be extended to improve the SNR of the GNSS signal. However, it can be seen from the above analysis that the extension of the coherent integration time is prevented by the oscillator noise. Therefore, it is necessary to analyze the SNR gain and the performance benefits of acquisition and tracking that can be obtained after the coherent integration extension with LEO assistance.

### 4.1. Coherent Integration Time Parameter Selection and the SNR Gain

The selection of coherent integration time is a key part of the design of GNSS receivers. It is the result of a compromise between various factors. On the one hand, to reduce noise and improve SNR, the integration time should be as long as possible. On the other hand, the integration time cannot be too long due to the attenuation of clock noise according to the analysis above. The selection of coherent integration time must consider both the noise and clock dynamic impact of the receiver. The coherent integral gain can be expressed as:

(11) $$G_{coh}=10lg\left(B_{pd}T_{coh}\right)$$

where $B_{pd}$ and $T_{coh}$ indicate the signal pre-detection bandwidth and coherent integration time, respectively. As the coherent integration time increases, the oscillator noise of the receiver accumulates to produce a frequency deviation, which causes the attenuation of the coherent integral gain. To avoid attention, non-coherent accumulation is adopted following coherent integration. The gain of non-coherent accumulation is as follows:

(12) $$G_{nc}=10lgN_{nc}-L_{SQ}$$

where $N_{nc}$ represents the number of non-coherent accumulations. $L_{SQ}$ is squaring loss, which exists because the mean of the output noise is not equal to zero due to the squaring operation. Squaring loss $L_{SQ}$ can be expressed as follows:

(13) $$L_{SQ}=SNR_{coh}-SNR_{SQ}$$

where $SNR_{coh}$ is the SNR of the coherent integration signal, and $SNR_{SQ}$ is the SNR of the non-coherent integration signal using the same duration, which is defined by:

(14) $$SNR_{SQ}=\frac{\pi }{\gamma \left(4-\pi \right)}{\left(e^{-\gamma /2}\left[\left(1+\gamma \right)I_{0}\left(\gamma /2\right)+\gamma I_{1}\left(\gamma /2\right)\right]-1\right)}^{2}.$$

**Figure 8:**
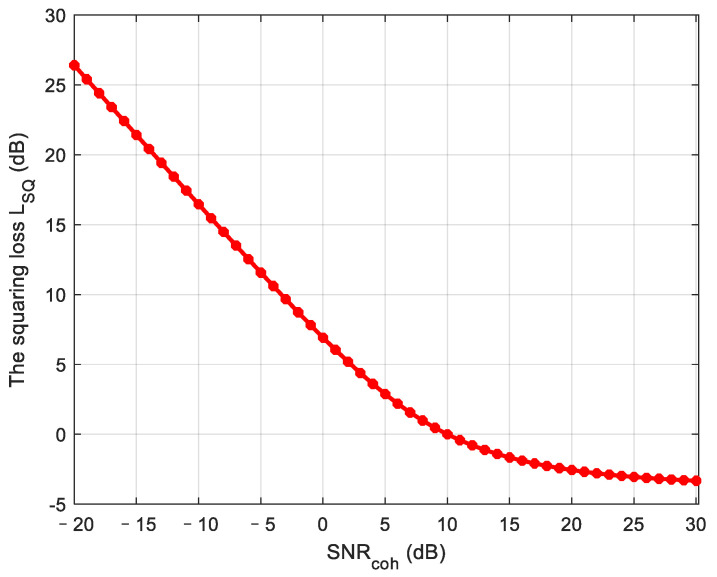
The squaring loss of non-coherent integration.

In the equation above, γ is defined as $P^{2}/2\sigma_{n}^{2}$, *P* is a given autocorrelation amplitude, $\sigma_{n}^{2}$ is the noise variance. $I_{n}\left(\cdot \right)$ stands for n order modified Bessel function. Figure 8 shows squaring loss in non-coherent integration. It can be seen that a higher SNR before non-coherent accumulation corresponds to a lower squaring loss.

**Figure 9:**
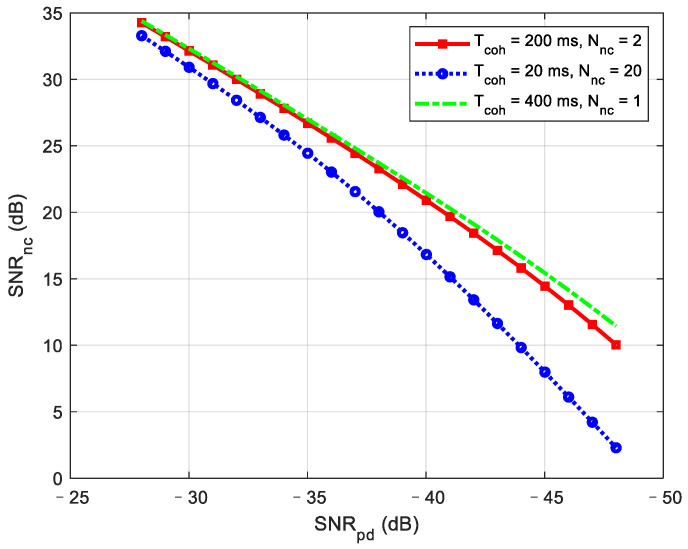
SNR performance under different integration combinations.

Using the equations above, we compare the SNR gain under different combinations of coherent integration and non-coherent integration. The noise bandwidth before the correlator is set to 2046 MHz. The range of the pre-detection SNR is from −48 to −28 dB. According to the analysis in Section 3.3, the total integration time is selected as 400 ms considering the influence of TCXO mentioned above. The simulation results are shown in Figure 9. It is obvious that a longer coherent integration time results in greater SNR gain. However, due to the influence of non-coherent loss, the gain of the 200 ms coherent integration time with two non-coherent integrations is almost the same as the direct 400 ms coherent integration, even in the ultra-low SNR stage. Therefore, it is suggested to use 200 ms coherent integration with two non-coherent combinations instead of 400 ms coherent integration, considering the frequency search step reduced along with the longer coherent integration.

### 4.2. Acquisition Performance with LEO Assisted

Using LEO to assist GNSS signal acquisition, one benefit is the reduction of the signal search range. The total number of search units in the two-dimensional search range of code phase and frequency can be expressed as:

(15) $$N_{cell}=\left(\frac{2f_{unc}}{f_{bin}}+1\right)\frac{2t_{unc}}{t_{bin}}$$

where $f_{unc}$ and $t_{unc}$ are the uncertain ranges of the signal frequency and code phase, respectively. The searching step in the frequency and code domain are $f_{bin}$ and $t_{bin}$, respectively. It can be seen from Equation (15) that the signal acquisition time could be reduced when the number of searches in the uncertain range is small. The search step is typically set to 1/2 chip in the code phase. The relationship between the receiver position error Z and the code phase search number of times N is as follows:

(16) $$N=ceil\left(\frac{2\times Z}{\left(1/1023\right)\times 10^{-3}\times C}\right).$$

The typical standalone receiver’s code search range for a single GPS satellite is 1023 chips. However, in the LEO-assisted acquisition method, according to the pseudo-range error of the Iridium satellite is [−1000 m,1000 m], the chip deviation caused by the initial positioning error is about six chips, which means the chip search efficiency can be increased by about 170 times.

**Figure 10:**
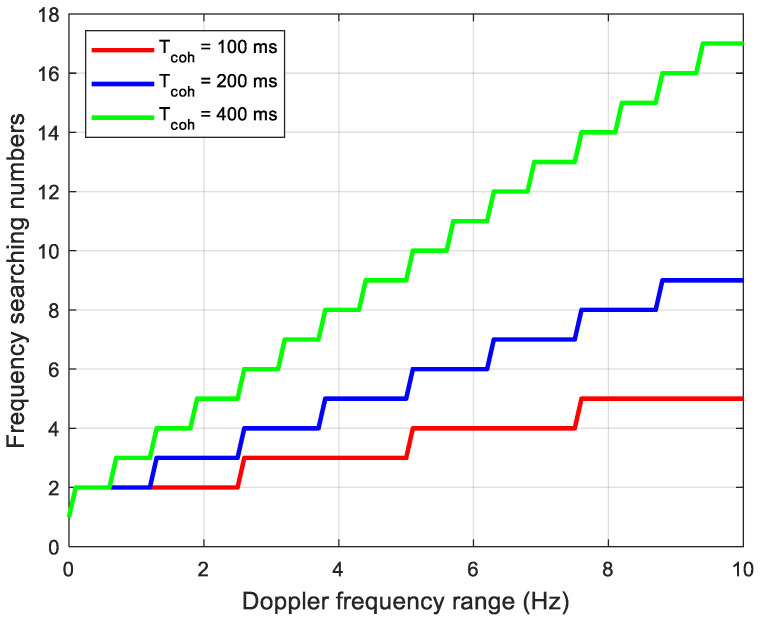
Frequency searching numbers according to the frequency range.

Concerning frequency searching, a different coherent integration time corresponds to different frequency steps. According to the analysis of Section 3, in the LEO positioning-assisted GNSS receiver structure, the frequency error mainly comes from the accuracy of the receiver clock. For commonly used TCXO, the frequency error range is (−10 Hz, 10 Hz). In addition, the stability of the clock limits the coherent integration time to 400 ms. The frequency search step is 2.5 Hz corresponding to a coherent integration time of 200 ms. Figure 10 shows the relationship between Doppler shift deviation and frequency search times under different coherent integration times. It can be seen that a longer integration time leads to larger numbers of frequency searching. Therefore, it is necessary to consider the balance between the SNR gain and the time required to search the entire range of frequency uncertainty. Compared with 200 ms coherent integration, the frequency search step is halved in 400 ms coherent integration, resulting in quadrupled search time. On the other hand, the frequency error caused by the instability of the receiver and the satellite clock increases as the coherent integration time increases. The coherent integration attenuation increases accordingly. Therefore, as a result of compromise, the coherent integration time is set to 200 ms in the implementation.

**Figure 11:**
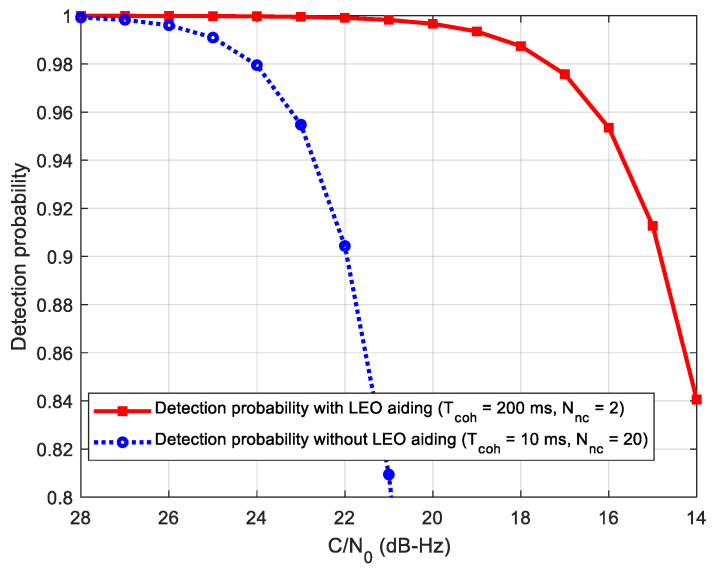
Acquisition performance under different carrier-to-noise ratio (C/N_0_).

The LEO-assisted GNSS acquisition method not only can improve the efficiency of acquisition but also can improve the acquisition sensitivity. As we know, a long coherent integration time can be used to improve the detection probability in weak signal environments. In the acquisition, the longest coherent integration is limited to 10 ms for a standalone receiver because of the navigation bit reversal. The non-coherent integration method could be used to improve the SNR furtherly but with a square loss due to the analysis above. However, the LEO-assisted acquisition can extend the coherent integration time with aiding of the received navigation information. On the other hand, according to the preliminary estimation of the Doppler and code phase obtained from LEO assistance, a higher false alarm probability can be set to improve the detection probability. Due to non-coherent integration being used in the acquisition, the results of correlation obey the central chi-square distribution when there is no signal. The probability density function (pdf) of the chi-square distribution is as follows:

(17) $$f_{n}\left(z\right)=\frac{z^{\frac{k}{2}-1}e^{-\frac{z}{2}}}{2^{\frac{k}{2}}\;\Gamma \left(\frac{k}{2}\right)}$$

where Γ(·) is the Gamma function, and *k* represents the degrees of freedom. Here, *k* is twice the non-coherent number. Assuming that the false alarm probability $P_{fa}$ is 0.5%, the threshold $V_{t}$ and detection probability can be calculated. Figure 11 gives the comparison of the signal detection probability of the two methods, where the coherent integration time is set to 200 ms with two non-coherent integrations according to the analysis above. It can be seen from this figure that the detection probability can reach 91% at C/N_0_ = 15 dB-Hz using the proposed method, which combines 200 ms coherent integration and two non-coherent integrations. However, the detection probability with 91% of the standalone receiver is at 22 dB-Hz. That means that the LEO-assisted acquisition has about 7 dB gain with regard to the detection probability.

### 4.3. Tracking Sensitivity with LEO Assisted

**Figure 12:**
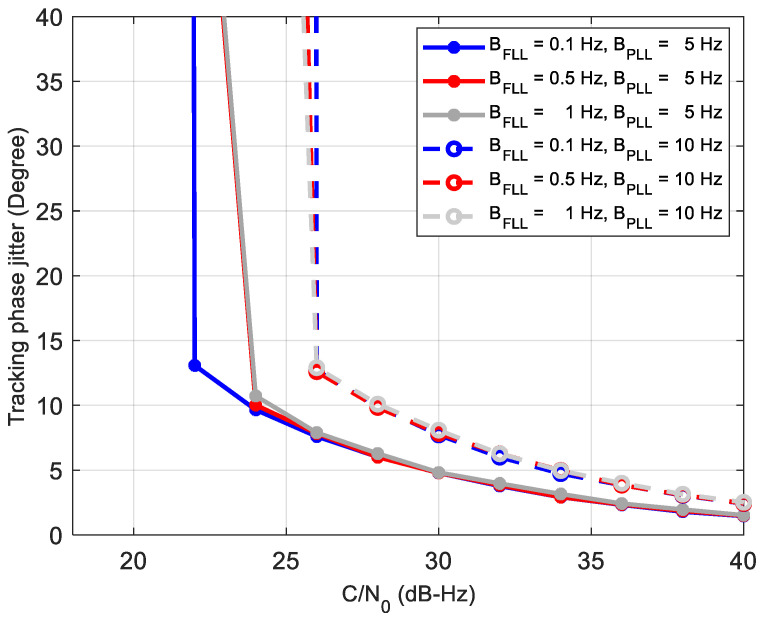
Phase jitter using the 2nd-order FLL assisted 3rd-order Phase-locked loop (PLL).

For comparison, the performance of the third-order PLL filter with second-order FLL assist [3] in a standalone receiver is analyzed by simulation firstly. The coherent integration time is set to be 20 ms due to the navigation bit reversal. The discriminator of the PLL is atan (*Q_P_/I_P_*), and that of the FLL is atan2(cross,dot)/Δt. Figure 12 shows the phase jitters of the tracking loop under different coefficients. From the figure, we can see that the phase jitter and tracking sensitivity are basically dependent on the bandwidth of the PLL. When the PLL bandwidth is 10 Hz, it loses lock at 26 dB-Hz, regardless of the FLL bandwidth. In contrast, the PLL loop with 5 Hz has a smaller phase jitter and can track the 22–24 dB-Hz signal, which is almost consistent with the results of the classical reference [3]. Thus, in the 2nd order FLL-assisted 3rd-order PLL structure, the FLL can reduce the impact of the dynamic. However, when the dynamic is eliminated, the bandwidth of the PLL decides the tracking sensitivity. Moreover, a narrower PLL bandwidth induces a smaller phase jitter and a better tracking sensitivity.

To solve the problem of the loss of lock under an ultra-low SNR condition, such as wideband jamming and signal blocked navigation, we propose the LEO-assisted GNSS tracking method in Figure 3. Since the dynamic is eliminated in the new method and the GNSS ephemeris can be received from the LEO, the receiver can extend the coherent integration time and reduce the loop bandwidth to track the ultra-weak signal. With the known ephemeris, a frequency-locked loop can be used instead of the PLL to improve the tracking sensitivity furtherly. In FLL tracking, the frequency jitter caused by mechanical vibration and Allan variance can be ignored [26]. The frequency jitter $\sigma_{tFLL}$ mainly comes from thermal noise.

**Figure 13:**
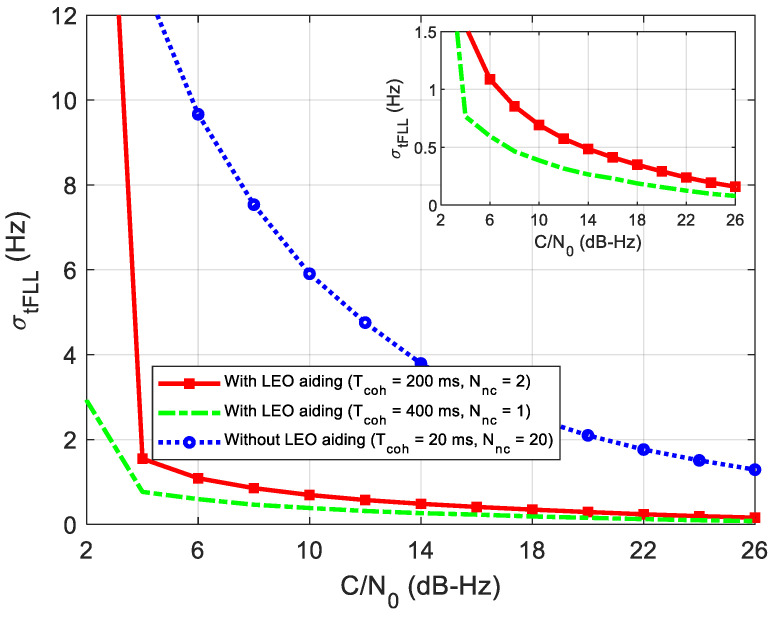
FLL frequency jitter caused by thermal noise.

Figure 13 gives the simulation results of the frequency jitter caused by the thermal noise. It can be seen that frequency jitters keep decreasing as the C/N_0_ increases. A long coherent integration time of 400 or 200 ms can greatly reduce the frequency jitter compared to that of the 20 ms. Nevertheless, the combination of 200 ms coherent integration and two non-coherent integrations can achieve almost the same performance as the 400 ms coherent integration. The result is coincident with the SNR analysis above. In addition, we can also figure out that the proposed method can track weak signals of 4 dB-Hz, just considering the thermal noise.

## 5. Test Results

To verify the performance of the LEO augmented acquisition and tracking structure, we use the GPS signal software simulator to generate the GPS IF signal with the augmentation of LEO. A software receiver [27] is used to acquire and track the signal. The input information of the signal simulator includes an LEO-assisted position error, frequency error, GPS satellite trajectory, start time and duration, ephemeris, almanac, and receiver’s oscillator model. The output is a digital intermediate frequency signal. The satellite constellation uses the real GPS constellation on 21 December 2014. The sampling frequency is set to be 5 MHz, and the intermediate frequency is 0 Hz. The white noise coefficient $h_{0}$ and random walk noise coefficient $h_{-2}$ of the receiver’s oscillator are set to $2\times 10^{-19}$ and $2\times 10^{-20}$, respectively. The sensitivity performance of the proposed LEO-assisted GNSS signal acquisition and tracking is tested experimentally.

### 5.1. Acquisition Performance Test

**Figure 14:**
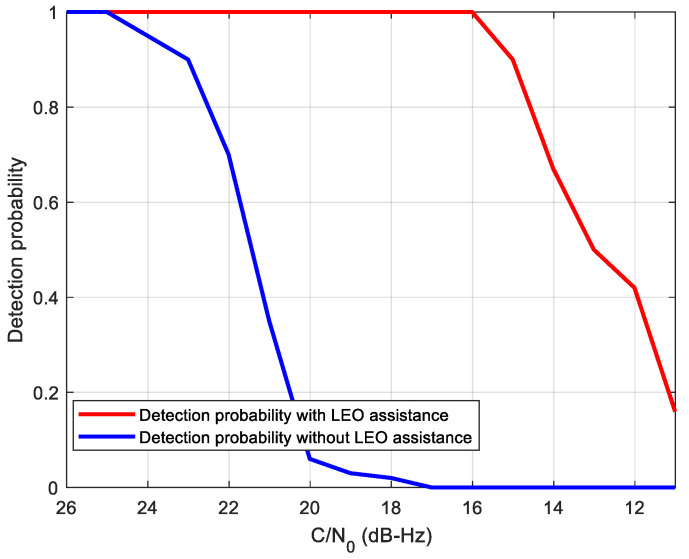
Acquisition performance comparison between LEO-assisted and unassisted method.

Firstly, we compare the sensitivity performance of the proposed acquisition method with the standalone conventional half-bit acquisition method. According to the analysis in the last section, we select a coherent integration time of 200 ms with two non-coherent combinations for the LEO-assisted method. The total integration time of the two methods is set to 400 ms. A statistical analysis of 100 times detection probability simulations is shown in Figure 14. It can be seen that the proposed acquisition method can achieve a detection probability of 90% even at C/N_0_ = 15 dB-Hz, which is consistent with the performance analysis result in Figure 11. It also can be seen that the proposed method outperforms the traditional method by about 8 dB. According to the performance analysis of Section 4, the 8 dB gain mainly comes from two aspects. First, without the navigation bit, the longest integrated time in the traditional method is limited to 10 ms, which means 200 ms integration time in total, resulting in 3 dB loss in C/N_0_ gain. Moreover, the C/N_0_ in the proposed method is 10 dB higher than that in the traditional method before non-coherent integration, which causes about 5 dB squaring loss in the weak C/N_0_. Therefore, the profits of the assisted acquisition method on sensitivity is consistent with the analysis above.

### 5.2. Tracking Performance Test

**Figure 15:**
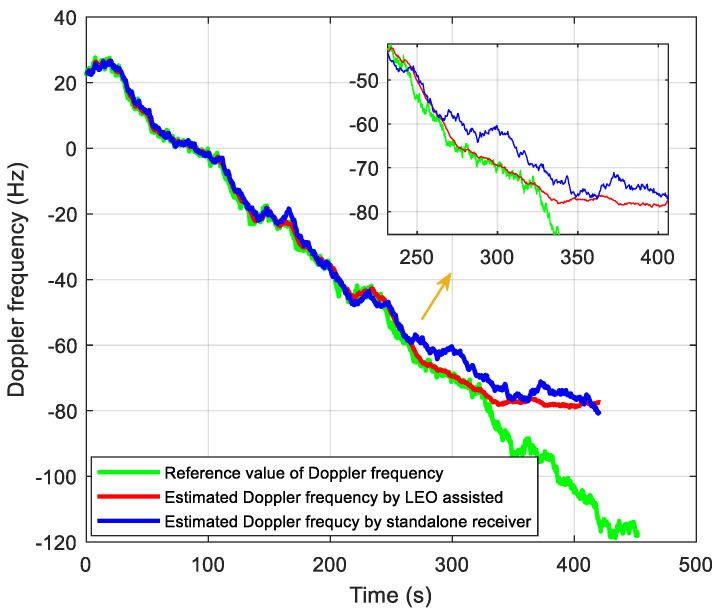
Performance comparison of frequency tracking.

**Figure 16:**
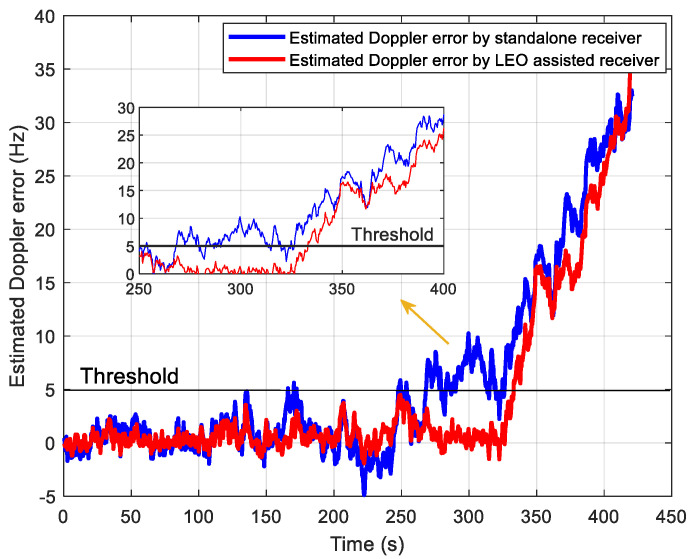
Performance comparison of estimated Doppler error.

In the signal tracking performance test, we generate a set of intermediate frequency data whose C/N_0_ decreases from 21 to 1 dB-Hz with a speed of 2 dB per 40 s. The traditional tracking method without assisted information is realized for comparison, where the total integration time is set to 400 ms, including a 20 ms coherent integration time and 20 non-coherent integrations. The tracking results are shown in Figure 15 and Figure 16. Figure 15 shows the estimated Doppler with and without the LEO assistance. Moreover, Figure 16 shows the estimated Doppler errors relative to the reference value of the Doppler frequency. When the estimated Doppler error exceeds the threshold of 5 Hz, it is considered to lose lock. We can see that the proposed tracking method can track up to 320 s, corresponding to C/N_0_ at 5 dB-Hz. It is consistent with the performance analysis in Figure 13. For the traditional method, it can be seen the estimated Doppler error exceeds 5 Hz at 250 s (9 dB-Hz), resulting in a loss of lock. Such a high-sensitivity performance in the standalone receiver is mainly from the DA-based FLL structure mentioned above. Thus, the tracking sensitivity of LEO-assisted method is improved by about 4 dB compared with the unassisted method.

## 6. Discussion and Conclusions

With the rapid development of LEO satellites, the joint positioning method of LEO and GNSS has been extensively studied. However, as in most studies, it just stays at the level of observed value fusion. To improve the performance of GNSS receivers under ultra-weak signal conditions, we propose a novel receiver structure with an LEO-augmented GNSS system, in which LEO-assisted GNSS signal acquisition and tracking methods are adopted. By utilizing the LEO satellite to realize Doppler positioning, this method initializes the position and time of the GNSS receiver and effectively reduces the acquisition and tracking search range of the receiver. Through the ephemeris information broadcasted by the LEO satellite, GNSS receivers can eliminate the impact of bit reversal and increase the coherent integration time. We carry out theoretical analysis and simulation experiments on the proposed acquisition and tracking scheme. Results show that the acquisition efficiency is improved by 170 times, acquisition sensitivity is improved by 8 dB, and the tracking sensitivity is improved by 4 dB compared with the traditional standalone receivers. Concerning LEO positioning, we only use the existing Iridium Doppler positioning as an example. Our proposed LEO-augmented GNSS receiver structure is based on the LEO positioning. The information LEO provided is independent of specific LEO constellations. Moreover, we choose the positioning error range within 1 km, which is wide enough. The only difference is that the LEO is assumed to transit GNSS ephemeris in this work. Thus, the presented results are generic with other LEO constellations if the LEO satellite is transmitting the GNSS ephemeris.

This paper mainly discusses the high-sensitivity benefits of an LEO-assisted GNSS receiver, which is considered to be used in the ultra-weak signal environment, such as the strong wideband jamming background. Our next research work will focus on the performance of the structure under high-precision and high-dynamic applications. For high-precision applications, the PLL or FLL-assisted PLL should be used to get the carrier phase. With regard to the high dynamic applications, the GNSS/INS coupled system will be considered to reduce the impact of the receiver motion. In addition, with the development of LEO positioning technology, LEO satellite constellations with multiple satellites can effectively improve the initial positioning and timing accuracy, which means that the augmented effect will be further increased.

## Data Availability

Data sharing not applicable.
